# Direct carotid-cavernous fistula: an atypical presentation

**DOI:** 10.11604/pamj.2019.34.204.19445

**Published:** 2019-12-18

**Authors:** Yousra Ajhoun, Adil El Khoyali, Ismail Aissa, Nisrine Laaribi, Yassine Mouzari, Karim Reda, Abdelbarre Oubaaz

**Affiliations:** 1 Department of Ophthalmology, Military Hospital Mohammed V, Faculty of Medicine and Pharmacy of Rabat, Mohammed V University, Rabat, Morocco; 2 Department of Anesthesiology and Intensive Care, Military Hospital Mohammed V, Faculty of Medicine and Pharmacy of Rabat, Mohammed V University, Rabat, Morocco

**Keywords:** Direct carotid-cavernous fistula, insidious onset, craniofacial trauma

## Abstract

Posttraumatic carotid-cavernous fistula (CCF) is a very rare complication that can occur in patients with craniomaxillofacial trauma. It is defined by abnormal communication between arteries and veins located in the cavernous sinus. CCFs can be divided into two groups: direct, which are usually post traumatic and classically with a high flow and acute onset of symptoms. On the other hand, indirect CCFs are in the most of cases idiopathic and typically insidious of onset. The aim of the present case report is to describe an atypical presentation of direct CCF characterized by the insidious onset of symptoms with the goal to think about this rare complication and so not to delay the treatment which is an emergency in this case.

## Introduction

CCF is a very rare complication that can occur in patients with craniomaxillofacial trauma [1]. It is defined by abnormal communication between arteries and veins located in the cavernous sinus. CCF can be classified into two groups direct and indirect. Classically direct CCF is a high flow lesion with acute onset of symptoms [2]. However direct CCF with insidious onset of symptoms still uncommon.

## Patient and observation

**Figure 1:**
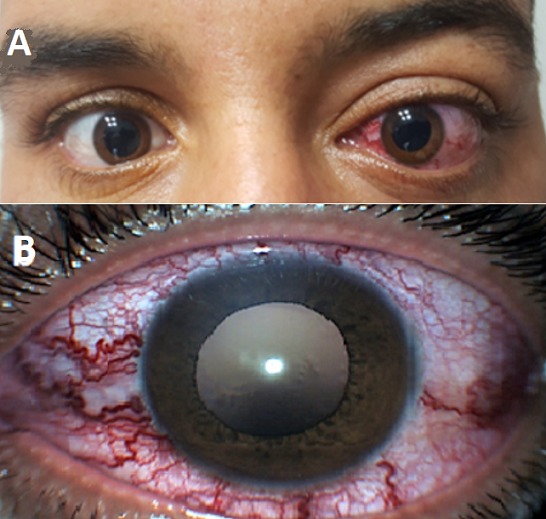
Facial photography demonstrating; (A) left axil proptosis; (B) dilated episcleral vessels with corkscrew-like aspect on the left eye

**Figure 2:**
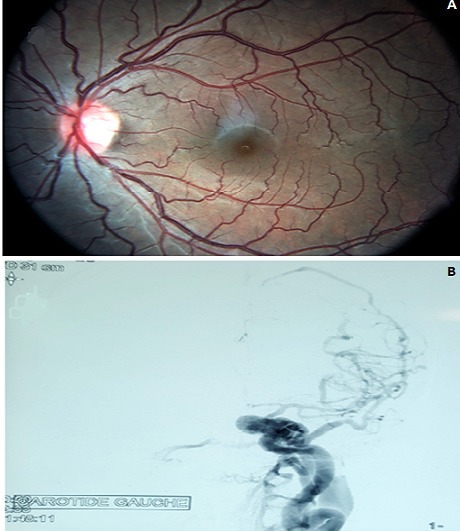
(A) left eye fundus photography revealing venous engorgement and tortuosity; (B) carotid arteriography showing direct CCF with opacification of the inferior petrosal sinus; the superior ophthalmic vein is thrombosed and slightly opacified

We report the case of 27 year-old man who suffered left cranio-facial trauma, caused by motorcycle accident. Consequently the patient presented mandibular fracture which was fixed. four months after the accident, the patient consult for left red eye, sensation of progressive proptosis and the hearing of buzzing sound with progressive intensity, without pain nor visual impairment. The ophthalmologic examination was notable in the left eye for axil, reducible exophthalmos with thrill on palpation (Figure 1 A), and dilated episcleral vessels (corkscrew-like aspect) (Figure 1 B). Anterior segment examination was unremarkable. Intra ocular pressure was normal. Fundoscopic examination found venous engorgement and tortuosity with normal optic disc aspect (Figure 2 A). Ophthalmologic examination in the right eye was normal.

**Figure 3:**
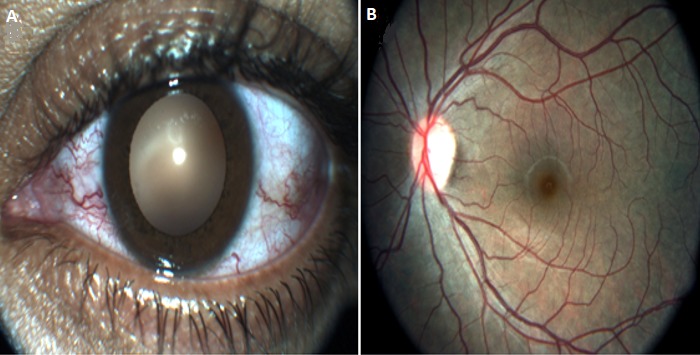
Left eye photography (A) and fundus photography (B) regression of the conjunctival vessels dilatation as well as retinal venous engorgement and tortuosity after fistula embolization

After such clinical findings, the diagnosis of CCF was the most probable. We completed with head arteriography. As expected this test revealed the presence of left CCF with shunt onthe C3-C4 segment of the internal carotid artery (ICA).The venous drainage was via posterior into the inferior petrosal sinus, lateral into the sphenoparietal sinus and the superior sagittal sinus, inside into the contralateral cavernous sinus and anterior the thrombosed superior ophthalmic vein was slightly opacified (Figure 2 B). Endovascular approach was indicated. Our patient undergone fistula embolization with coils after arterial catheterization. After 20 days we have noticed regression of exophthalmos, conjunctival vessels vasodilatation as well as retinal venous engorgement and tortuosity (Figure 3 A,B).

## Discussion

CCFs are rare trauma complication [1]. Incidence rates range around 0.2% in all cases of head and face trauma [3]. Barrow classified CCFs into two groups: direct CCFs (type A) correspond to a connection between the internal carotid artery and the ipsilateral cavernous sinus, indirect CCFs are divided into 3 types: type B results from a connection between meningeal branches of the internal carotid and the sinus, type C is characterized by communication between meningeal branches of the external carotid and the sinus and finally type D is characterized by connections between meningeal branches of the internal and external carotid and the sinus [4].

Direct CCFs are usually post head trauma, secondary to motor vehicle accidents or penetrating injuries, they may also be caused by rupture of intra-cavernous aneurysm or head surgery [2,5,6]. Our patient presented direct CCF caused by craniofacial trauma secondary to motor vehicle accident. The clinical symptoms of direct CCFs are classically abrupt in onset with typically rapid progression [2, 7, 8], necessitating urgent treatment. In contrast indirect CCFs are in the most of cases idiopathic and tend to be more insidious in onset [9]. Indeed the severity of symptoms is determined by venous return capacity and by quantity and speed of the blood flow [10]. In most cases there is a mixed anterior and posterior drainage, anterior into the ophthalmic veins and posterior into the petrosal sinuses. The most dramatic symptomatology is seen in case of anterior drainage. The orbital manifestations are less severe when the venous drainage is posterior [11]. Our patient was diagnosed with direct CCF but with insidious onset of symptoms which is an uncommon presentation, this situation is explained by the presence of thrombosed superior ophthalmic vein, consequently the fistula drains essential into the inferior petrosal sinus.

The main symptoms of CCFs include orbital bruit which can be heard by the patient and objectively determined by the physician, proptosis, chemosis , arterialization of the episcleral veins with the typical corkscrew-like aspect, eyelid edema, diplopia, visual impairment with different degrees and elevated intra ocular pressure. The ophthalmologic examination may find also dilation of retinal veins, intraretinal hemorrhages, mild optic disc swelling and even non-rhegmatogenous retinal detachments and choroidal detachments [12]. Our patient presented; axilreducible exophthalmos with thrill on palpation, dilated episcleral vessels (corkscrew-like aspect) with retinal venous engorgement and tortuosity as also found in the literature.

Diagnosis is based on the angiographic study, this exam allows the correct lassification of each case and also determine the treatment strategy [10]. In cases of direct CCFs with progressive symptoms, visual loss occurs in nearly 90% of untreated patients which stress the necessity of prompt treatment [12]. Trans-arterial embolization using detachable balloons is considered to be as the treatment of choice for direct CCFs [13, 14]. However, an alternative for balloon embolization are detachable platinum coils, either as a primary agent or in combination with balloons [12]. Conservative treatment is indicated for small, asymptomatic, or stable fistulae, since these may close spontaneously.

## Conclusion

Throughout our case report we described an atypical presentation of direct CCF characterized by the insidious onset of symptoms with the goal to think about this rare complication and so not to delay the treatment which is an emergency in this case.

## Competing interests

The authors declare no competing interests.
